# Prevalence and antibiotic resistance profile of Shiga toxin-producing *Escherichia coli* isolated from diarrheal samples

**DOI:** 10.18502/ijm.v12i4.3931

**Published:** 2020-08

**Authors:** Farzad Esavand Heydari, Mojtaba Bonyadian, Hamdallah Moshtaghi, Masoud Sami

**Affiliations:** 1 Department of Health and Food Quality Control, Shahrekord University, Shahrekord, Iran; 2 Department of Health and Food Quality Control, Institute of Zoonoses Research, Shahrekord University, Shahrekord, Iran; 3 Department of Food Sciences and Technology, School of Nutrition and Food Science, Isfahan University of Medical Sciences, Isfahan, Iran

**Keywords:** Shiga-toxin, *Escherichia coli*, Antibiotic resistance, Diarrhea, Virulence genes

## Abstract

**Background and Objectives:**

Enterohemorrhagic *Escherichia coli* (EHEC) causes bloody and non-bloody diarrhea, intestinal infection and extraintestinal complications in humans. This study aimed to isolate and evaluate the prevalence of *E. coli* O157: H7 and other Shiga toxin-producing *E. coli* (STEC) and identify the virulence genes (*stx1, stx2, hly* and *eaeA*) from patients with diarrhea. Also, the antibiotic resistance profile of the isolated strains was evaluated.

**Materials and Methods:**

A total of 100 stool samples were collected from patients with acute diarrhea referring to the hospital and clinics in Isfahan County, Iran. Phenotypic tests and PCR assay were used for detection of *E. coli* O157: H7 and other Shiga toxin-producing *E. coli*. The presence of virulence genes (*stx1, stx2, hly* and *eaeA*) were identified by PCR. The antibiotic resistance profile of the isolates was determined using the agar disk diffusion method. The results were analyzed descriptively by Sigma stat version 4 software.

**Results:**

Seventy - eight out of 100 samples (78%) were contaminated with *E. coli. E. coli* O157 was isolated from five samples (6.4%), of which only two strains (2.56%) were identified as *E. coli* O157: H7. According to the results, out of two *E. coli* O157: H7 isolates, one (50%) isolate contained *eaeA* and two isolates (100%) contained *Stx1, Stx2, hlyA* genes. Out of three (3.84%) *E. coli* O157: HN, one of the isolate (33.3%) contained *stx1* and, two isolates (66.7%) were positive for *hlyA* genes. Also, the results revealed that six strains (7.69%) were non-O157: H7 STEC, of which two isolates (33.3%) contained *stx1* and four isolates (66.7%) were positive for *stx2* and *hlyA* genes. The results of antibiogram tests revealed that all of the STEC isolates (100%) were sensitive to imipenem followed by kanamycin, gentamicin and nitrofurantoin (91%). High resistance (54.5%) to ampicillin and ciprofloxacin was observed among the STEC isolates.

**Conclusion:**

The results of the current study showed that although the prevalence of *E. coli* O157: H7 was low among patients with diarrhea, the other STEC strains with relative resistance to antibiotics are more prevalent.

## INTRODUCTION

According to released reports, diarrhea accounts for more than half of global foodborne diseases. *Escherichia coli* is one of the most important microorganisms causing diarrhea around the world (1). The six main categories include enteropathogenic *E. coli* (EPEC), enterotoxigenic *E. coli* (ETEC), enteroinvasive *E. coli* (EIEC), enteroaggregative *E. coli* (EAEC), enterohemorrhagic *E. coli* (EHEC or STEC), diffuse adhering *E. coli* (DAEC) was investigated. Shiga toxin-producing *E. coli* (STEC) particularly *E. coli* O157: H7, is one of the newly emerging pathogens that causes acute gastroenteritis and also bloody diarrhea. Enterohemorrhagic *E. coli* (EHEC) is one of the subtypes of STEC characterized by positive *stx/eae* and the ability to cause severe diseases such as HUS in humans. Shiga toxins prevent protein production in the host cell and cause cell killing. The *stx1* and *stx2* genes are the most important virulence factors of isolated EHEC. Studies show that virulence in humans and animals is higher in the presence of *stx2* than *stx1* (2). The *eae* gene codes for an extracellular protein called intimin, this protein is essential for binding bacteria to host enterocytes. Other pathogenicity factors include hemolysin, which acts as a cytolysin on eukaryotic cells and is coded by the *Hly* gene. *E. coli* O157: H7 is the most common cause of HUS, also other subtypes of non-O157 EHEC can cause HUS (3, 4). Approximately, 3–9% of STEC infections lead to HUS, systemic complications, and death (5). Contamination of foods such as minced meat, raw milk, vegetables and even water with STEC are the most important sources of human infection in rural areas (6, 7). An epidemiological study shows that more than 30% of the cows are the asymptomatic carriers for *E. coli* O157: H7 (8). It also causes mortality in 3–5% of patients and the long-term complications in 30% of patients (9). The low infectious dose of O157: H7 EHEC (10–100 CFU) increases the virulence and risk of infectivity (10). In EHEC infection, antibiotic therapy results in the release of verotoxin and the death of bacteria (9). To reduce the risk of STEC transmission from animals to a human and better understanding of STEC transmission and pathogenicity, different analytical methods are needed. Also, recognizing the similarities and differences among strains (focusing on the type of intimin and Shiga-like toxin genes) isolated from human infection and non-human samples may result in lowered infection risk (11). Previous studies have revealed the high incidence of antibiotic-resistant in the isolates of EHEC to cephalothin (92.30%) (12). Some results showed the high resistance rates of STEC strains against the commonly used antimicrobial agents such as ampicillin, amoxicillin, chloramphenicol, tetracycline, cotrimoxazole and nalidixic acid (12, 13).

This study aimed to isolate and evaluate the prevalence of *E. coli* O157: H7 and other Shiga toxin-producing *E. coli* (STEC) and identify the virulence genes (*stx1, stx2, hly* and *eaeA*) from patients with diarrhea. Also, the antibiotic resistance profile of the isolated strains was evaluated.

## MATERIALS AND METHODS

### Sampling and *Escherichia coli* identification.

A total of 100 fecal samples were collected from patients with acute diarrhea (38 males, 62 females) in Montazeri and Al-Zahra hospitals in Isfahan-Iran, from October 2017 to October 2018. Patient demographic characteristics including age and gender were recorded. To monitoring the infection side effects, patients were followed up for two months after sampling. Stool samples were cultured on MacConkey agar (SMAC, Merck, Germany) and sorbitol MacConkey agar (SMAC, Merck, Germany) and incubated at 37°C for 24 hours. Five lactose fermenting colonies were picked up from MCA and 5 non-sorbitol fermenting colonies (colorless) was transferred from SMAC into EC broth (Ibresco-life sciences) and incubated for 18 hours at 37°C. Phenotypic tests including Gram staining, Oxidase, Indole, Simon citrate, urease, and H_2_S production were performed to confirm *E. coli* (14). Isolated *E. coli* were maintained on Trypticase soy agar (TSA, Merck, Germany) until further experiments.

### DNA extraction.

The *E. coli* isolates were first cultured in Tryptone soy broth and incubated for 24 hours at 37°C. Then 1 ml of the culture was poured into a 1.5 ml tube and was centrifuged at 1200 rpm for five minutes. The supernatant was discarded and a bacterial suspension was prepared by adding the 200 μL of sterile distilled water to the sediment and mixed thoroughly. To extract DNA, the bacterial suspension was boiled for five minutes at 100°C to release the DNA and the aliquot was centrifuged in 1200 rpm for 3 min. The supernatant was used in the PCR as a DNA template. The concentration of DNA was determined by the spectrophotometer at 263 nm wavelength (15).

### Detection of virulence genes by PCR.

The standard strain of *E. coli* O157: H7 (ATCC 43895) was used as positive control and sterile distilled water as a negative control for PCR assay. PCR was performed according to the method described by Blanco M et al. (15). The set of primers including *rfbE* and H7 genes were used for identification of *E. coli* O157: H7 and the *stx1, stx2, eaeA, hlyA* for the presence of virulence genes (Table 1). PCR assay was carried out in a volume of 25 μL, so that 21 μL of Master Mix (CinnaGen, Iran), containing 400 mM of deoxy-nucleoside triphosphates, 0.05 U/μL of Taq DNA polymerase, 4 mM of MgCl_2_, 1 μL of each primer, and 2 μL of the DNA extracted. The PCR was carried out in 35 cycles using the thermocycler (Biorad, USA), (Table 2). The PCR products were electrophoresed at 100 V for 60 minutes, and stained with Safe-Red™ and photographed using a gel documentation system (Herolab, Weisloch, Germany) (15).

**Table 1.** Characterization of primers for identification of *E. coli* O157: H7 and virulence genes (Rey 2006), (16).

**Gene target**	**Primers**	**Oligonucleotide Sequence (5′-3′)**	**Fragment (bp)**	**Annealing Temp (°C)**	**Reference**
*rfbE*	O157-R	5′-CGTGGTATAGCTACTGTCACC-3′	259	58	21
	O157-F	5′-CGCTGAATGTCATTCGCTCTGC-3′			
*stx1*	*stx1*-F	ATA AAT CGC CAT TCG TTG ACT AC	180	48	24
	*stx1*-R	AGA ACG CCC ACT GAG ATC ATC			
*stx2*	*stx2*-F	TTA ACC ACA CCC CAC CGG GCA GT	524	55	25
	*stx2*-R	GGA TAT TCT CCC CAC TCT GAC ACC			
*eaeA*	EAE-R	5′-GCGGTATCTTTCGCGTAATCGC<C>-3′	775	50	26
	EAE-F	5′-GAGAATGAAATAGAAGTCG<T>-3′			
*flIcH7*	H7-R	5′-CAACGGTGACTTTATCGCCATTCC-3′	625	60	27
	H7-F	5′-GCGCTGTCGAGTTCTATCGAGC-3′			
*hlyA*	hlyAF	5′-CATCGGCTGTTATGCTGG-3′	513	56	Accession number: AP01848901
	hlyAR	5′-CATCCCAATGTTGCTGGG-3′			

**Table 2.** PCR condition using for identification of virulence genes in STEC strains.

**No**	**Step**	***rfbE* (O157)**	**H7 *(flic)***	***hly* and *eae***	***Stx1* and *Stx2***
1	Initial denaturation	94°C/4 min	94°C/4 min	94°C/4 min	94°C/4 min
2	Denaturation	94°C /30 sec	92°C /30 sec	94°C/45 sec	94°C/30 sec
3	Annealing	58°C/45 sec	62°C/50 sec	54°C/1 min	50°C/30 sec
4	Extension	72°C/45 sec	72°C/45 sec	72°C/45 sec	72°C/45 sec
5	Final extension	72°C/45 sec	72°C/45 sec	72°C/45 sec	72°C/45 sec

### Antimicrobial susceptibility test.

The antibiotic resistance profile of STEC isolates was determined using Mueller Hinton agar medium (Merck, Germany) using the disk diffusion method. The standard *E. coli* O157: H7 (ATCC 43895) was used as a control. Principles of the Clinical and Laboratory Standards Institute (CLSI 2018) guidelines were used for this purpose (17). Bacterial isolates were cultured in TSB and incubated for 24 hours at 37°C, the concentration of 0.5 McFarland of bacteria was used for antimicrobial resistance tests. Susceptibility of the isolates was tested against ampicillin (AM, 10 μg), trimethoprim-sulfamethoxazole (STX, 25 μg), kanamycin (KAN, 30 μg), gentamicin (GM, 10 μg), chloramphenicol (CLR, 30 μg), ciprofloxacin (CIPRS, 5 μg), ceftazidime (CAZ, 30 μg), nitrofurantoin (NIT, 300 μg), nalidixic acid (NAL, 30 μg), and imipenem (IPM, 10 μg), (Rosco, England). Antibiotic discs were placed on the cultured plate and incubated for 18–24 hours at 37°C. The growth inhibition zone was interpreted according to the standard range.

### Statistical analyzes.

The results were analyzed descriptively by Sigma Stat version 4 software.

## RESULTS

**Fig. 1.**
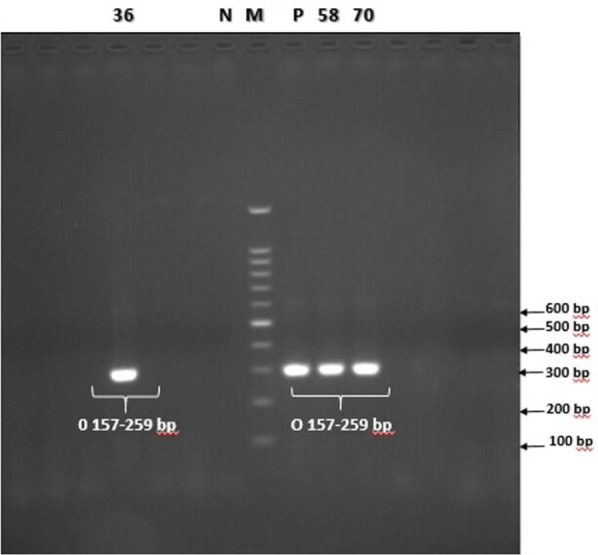
PCR products of the O157 gene *(rfbE)*. 36, 58 and 70 positive samples. M: 100 bp DNA ladder (Fermentas Co.), N (Negative control), P (Positive control)

**Fig. 2.**
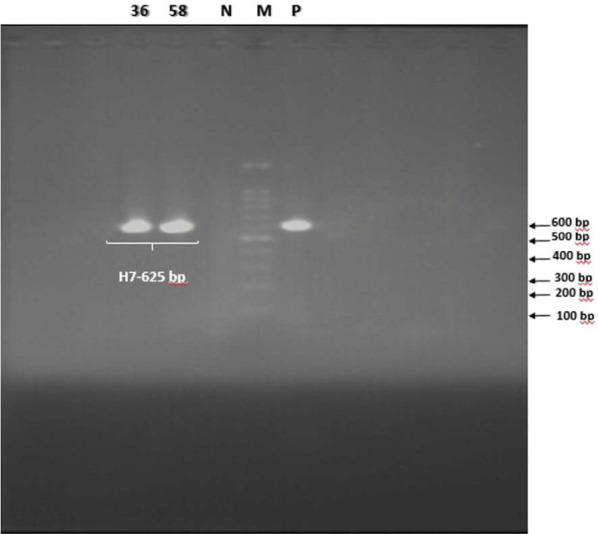
PCR products of the *H7* gene *(fliC)*. 36 and 58 positive samples, M: 100 bp DNA ladder, N (Negative control), P (Positive control).

**Fig. 3.**
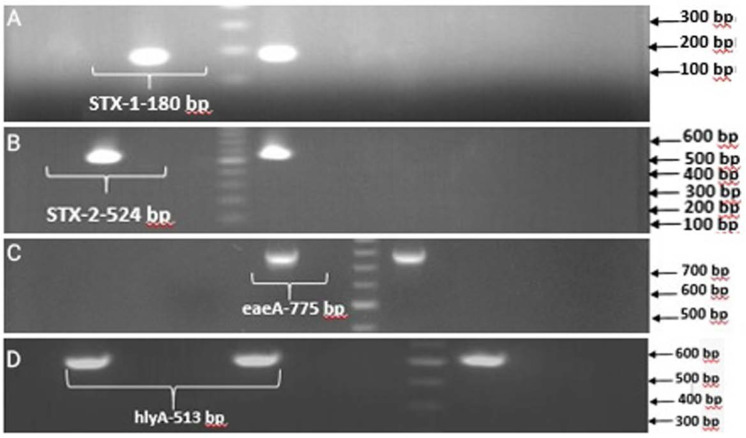
PCR products of the *stx1* (A), *stx2* (B), *eaeA* (C) and *hlyA* (D) genes.

A total of 100 fecal samples from patients with acute diarrhea were examined for the presence of Shiga toxin-producing *E. coli* (STEC). Fifty-one percent of the patients were female and more than 65% of the studied samples were non-bloody watery diarrhea and 30% bloody diarrhea. Seventy-eight out of 100 samples (78%) were positive for *E. coli* strains in stool culture. According to PCR results, five strains (6.41%) harbored the *rfbE* gene which identified as *E. coli* O157 (Fig. 1), but only two isolates (2.56%) were positive for *flicH7* gene (Fig. 2), which confirmed as *E. coli* O157: H7. Both of the *E. coli* O157: H7 isolates (100%) contained *Stx1, Stx2, hlyA* genes but only one isolate was positive for the *eaeA* gene. Also, 3 isolates (3.84%) were identified as *E. coli* O157: HN, of which, one isolate (33.3%) carried the *Stx1* gene and two (66.7%) isolates were positive for *hlyA* gene. According to the results, 6 isolates (7.69%) were identified as non-O157: H7 Shiga toxin-producing *E. coli*, of which, two isolates (33.3%) were positive for *stx1* gene only, while four isolates (66.7%) harbored a combination of *stx2* and *hlyA* genes. The presence of virulence genes in the STEC isolates is presented in Table 3, (Fig. 3). The infected patients with *E. coli* O157: H7 had diarrhea and vomiting and were hospitalized for three days. There were no signs of renal complications and HUS in studied patients.

**Table 3.** Prevalence of pathogenic subtypes and related virulence genes in STEC strains isolated from patients with diarrhea.

**Samples (No)**	**Subtypes**	**No. positive samples**	**Virulence genes**
Stool (100)	*E. coli*	78 (78%)	-
	None detected	22 (22%)	-
	*E. coli* O157: HN	3 (3.84%)	*Stx1*: 1 (33.3%)
			*Stx2*: -
			*eaeA*: -
			*hlyA*: 2 (66.7%)
	*E. coli* O157: H7	2 (2.56%)	*Stx1, Stx2, eaeA, hlyA*: 1 (50%)
			*Stx1, Stx, hlyA*: 2 (100%)
	*E. coli* non- O157: H7	6 (7.69%)	*Stx1*: 2 (33.3%)
			*Stx2, hlyA*: 4 (66.7%)
			*eaeA*: -

The results of antimicrobial resistance tests for the *E. coli* 0157: H7 and non-O157 strains are shown in (Table 4). According to the results, all of the isolates (100%) were sensitive to imipenem, gentamicin and nitrofurantoin followed by kanamycin and chloramphenicol (91%). The high antibiotic resistance to ampicillin and ciprofloxacin (54.5%), also, to trimetho-prim-sulfamethoxazole and ceftazidime (45.5%) were detected among STEC isolates.

**Table 4.** Antibiotic resistance profile of STEC strains isolated from patients with acute diarrhea.

**Antibiotics**	**No of resistance strains**
Ampicillin (AM/10 μg)	6 (54.5%)
Trimethoprim-Sulfamethoxazole (SXT/25 μg)	5 (45.5%)
Kanamycin (KAN/30 μg)	1 (9%)
Gentamicin (GM/10 μg)	0 (0%)
Chloramphenicol (CLR/30 μg)	1 (9%)
Ciprofloxacin(CIPRS/5 μg)	6 (54.5%)
Ceftazidime (CAZ/30 μg)	5 (45.5%)
Nitrofurantoin (NIT/300 μg)	0 (0%)
Nalidixic acid (NAL/30 μg)	4 (36.5%)
Imipenem (IMP/10 μg)	0 (0%)

## DISCUSSION

Bacterial agents cause about 24% of diarrhea and more than 70% of deaths due to diarrhea in children under five years (1). In medical diagnostic laboratories, colonies suspected to *Salmonella* and *Shigella* are usually considered as pathogens and Shiga toxin-producing *E. coli* is mostly neglected. In developing countries such as Iran, STEC is considered only in research centers but disregarded in treatment centers because of the high cost of the *E. coli* pathotyping diagnostic kits. Generally, the etiological factors of acute diarrhea in developing and developed countries are different. In developing countries, *Shigella* and *E. coli* are the dominant strains causing diarrhea (18). Many studies were conducted to identify the pathotype of EHEC in different parts of the world that were indicated the STEC strains are responsible for important human diseases including HUS, hemorrhagic colitis and thrombotic thrombocytopenic purpura. In severe cases, mortality is closely correlated with the presence of the virulence genes.

Patients with non-bloody diarrhea have a milder manifestation and are less likely to develop HUS and death. However, HUS is also reported in cases of non-bloody diarrhea (19). According to the findings of the current study, STEC strains such as *E. coli* O157: H7 and non-O157 strains, contributed to 11% of all infectious diarrheas which were able to produce shiga-like toxins. Previous studies show the low prevalence of *E. coli* O157: H7 strain in humans in developing countries. Accordingly, only 2% of patients in the present study were infected with this strain. This result is close to the other studies in Iran. Taghadosi et al. isolated five (1.3%) STEC strains from 395 diarrheal fecal samples in Iran (20). Similar studies in Kenya also indicated the low prevalence (0.2%) of *E. coli* O157: H7 in hospitalized diarrheic children (21). But in some countries high prevalence rate of STEC was reported, Ifeanyi in Nigeria (22) and Dambrozio in Brazil (2014) (23) identified EHEC pathotypes in 12.6% and 21.2% of children with diarrhea, respectively. Pourakbari et al. (2013) studied 50 children with diarrhea and 50 healthy ones referring to a medical center in Iran, *E. coli* was isolated in 90% of patients and 20% of healthy controls, also, EHEC strains were identified in 14% and 3% of patients and controls, respectively (24). In another study in Tabriz-Iran, 450 diarrheal and 150 non-diarrheal specimens were examined, *E. coli* was isolated from 140 diarrheal and 90 non-diarrheal samples, EHEC strains were isolated only from 17 diarrheal samples (19). The results of the present study revealed that non-O157 STEC strains were more prevalent (7.96%) in patients with gastroenteritis. Also, various studies in Iran showed that the prevalence of *E. coli* O157: H7 in humans and animals is lower than that of other STEC strains (25, 26).

There is worldwide concern about the appearance and rise of bacterial resistance to commonly used antibiotics. Previous studies revealed that enteric pathogens, as well as *E. coli*, were often resistant to multiple antibiotics. A comparison of the results of the present study with those of other ones, the resistance to ampicillin and susceptibility to imipenem were common in most of the studies and the difference was only in the reported prevalence rate. The extending of microbial resistance among the bacteria to the target antibiotics is attributed to the inappropriate use of antibiotics by clinicians and the easy accessibility of these agents, particularly in developing countries. According to the results of the present study, 54.5% of the STEC isolates were resistant to ampicillin and ciprofloxacin also, 45.5% to trimetho-prim-sulfamethoxazole and ceftazidime. Similar results, reported by researchers in Iran. In a study conducted by Bouzari et al. (2018) on patients with acute diarrhea, out of 102 *E. coli* isolates, 13 (12.7%) isolates were identified as EHEC, of which 76.9% were resistant to ampicillin, but 100% and 76.9% were susceptible to imipenem and gentamicin, respectively (2). Fazeli et al. revealed the high antibiotic resistance to amoxicillin (65.5%), tetracycline (58.6%) and sulfamethoxazole-trimethoprim (72.4%) in STEC isolated from diarrheal patients in Isfahan (27). A high prevalence antibiotic resistance of STEC strains isolated from raw milk and traditional dairy products to ampicillin (100%), gentamicin (100%) and tetracycline (96.87%) reported by Ranjbar et al. (28). Studies in other countries also show high levels of antibiotic resistance in *E. coli* isolates. In a study in Nigeria (2019), high antibiotic resistance to ampicillin (93.6%), ciprofloxacin (45.6%) and gentamicin (37.4%) were reported (29). The results also indicated common resistance of *E. coli* strains to ampicillin in most geographical regions, also, extended use of antibiotics resulted in the emergence of resistance genes and their transmission among strains.

In conclusion, the results of the current study showed that the frequency of *E. coli* O157: H7 strain is low in patients with acute diarrhea. Moreover, non-O157 STEC strains with high resistance to some common antibiotics such as cotrimoxazole, ampicillin, ciprofloxacin and ceftazidime are more frequent.
